# Long-Term Citizen-Collected Data Reveal Geographical Patterns and Temporal Trends in Lake Water Clarity

**DOI:** 10.1371/journal.pone.0095769

**Published:** 2014-04-30

**Authors:** Noah R. Lottig, Tyler Wagner, Emily Norton Henry, Kendra Spence Cheruvelil, Katherine E. Webster, John A. Downing, Craig A. Stow

**Affiliations:** 1 Center for Limnology Trout Lake Station, University of Wisconsin Madison, Boulder Junction, Wisconsin, United States of America; 2 U.S. Geological Survey, Pennsylvania Cooperative Fish and Wildlife Research Unit, Pennsylvania State University, University Park, Pennsylvania, United States of America; 3 Department of Fisheries and Wildlife, Michigan State University, East Lansing, Michigan, United States of America; 4 Department of Outreach and Engagement, Oregon State University, Tillamook, Oregon, United States of America; 5 Lyman Briggs College, Michigan State University, East Lansing, Michigan, United States of America; 6 School of Natural Sciences, Trinity College, Dublin, Ireland; 7 Department of Ecology, Evolution, and Organismal Biology, Iowa State University, Ames, Iowa, United States of America; 8 Great Lakes Environmental Research Laboratory, National Oceanic and Atmospheric Administration, Ann Arbor, Michigan, United States of America; University of Pennsylvania, United States of America

## Abstract

We compiled a lake-water clarity database using publically available, citizen volunteer observations made between 1938 and 2012 across eight states in the Upper Midwest, USA. Our objectives were to determine (1) whether temporal trends in lake-water clarity existed across this large geographic area and (2) whether trends were related to the lake-specific characteristics of latitude, lake size, or time period the lake was monitored. Our database consisted of >140,000 individual Secchi observations from 3,251 lakes that we summarized per lake-year, resulting in 21,020 summer averages. Using Bayesian hierarchical modeling, we found approximately a 1% per year increase in water clarity (quantified as Secchi depth) for the **entire population** of lakes. On an **individual lake** basis, 7% of lakes showed increased water clarity and 4% showed decreased clarity. Trend direction and strength were related to latitude and median sample date. Lakes in the southern part of our study-region had lower average annual summer water clarity, more negative long-term trends, and greater inter-annual variability in water clarity compared to northern lakes. Increasing trends were strongest for lakes with median sample dates earlier in the period of record (1938–2012). Our ability to identify specific mechanisms for these trends is currently hampered by the lack of a large, multi-thematic database of variables that drive water clarity (e.g., climate, land use/cover). Our results demonstrate, however, that citizen science can provide the critical monitoring data needed to address environmental questions at large spatial and long temporal scales. Collaborations among citizens, research scientists, and government agencies may be important for developing the data sources and analytical tools necessary to move toward an understanding of the factors influencing macro-scale patterns such as those shown here for lake water clarity.

## Introduction

Macrosystems ecology has emerged as a new ecology sub-discipline aimed at understanding ecosystem patterns and processes resulting from broad-scale environmental changes such as land use and climate change. This sub-discipline studies multi-scaled patterns and processes in biological, geophysical, and social components, and their interactions with each other and processes operating at finer and coarser scales [1]. One of the approaches that macrosystems ecologists are taking is integrating large datasets from a variety of sources. There may be many inherent challenges in assembling appropriate datasets for analysis in a macrosystems ecology context [2], however, we feel that well-established citizen monitoring programs can play an important role in macrosystems ecology, especially those that collect data across large spatial and temporal extents with standardized measurement, data entry, and quality control protocols [3]. The Christmas Bird Count is a well-known example of a citizen-based program (http://birds.audubon.org/about-christmas-bird-count), long recognized as a successful model of how to integrate citizens into meaningful science and conservation [4], [5].

**Figure 1:**
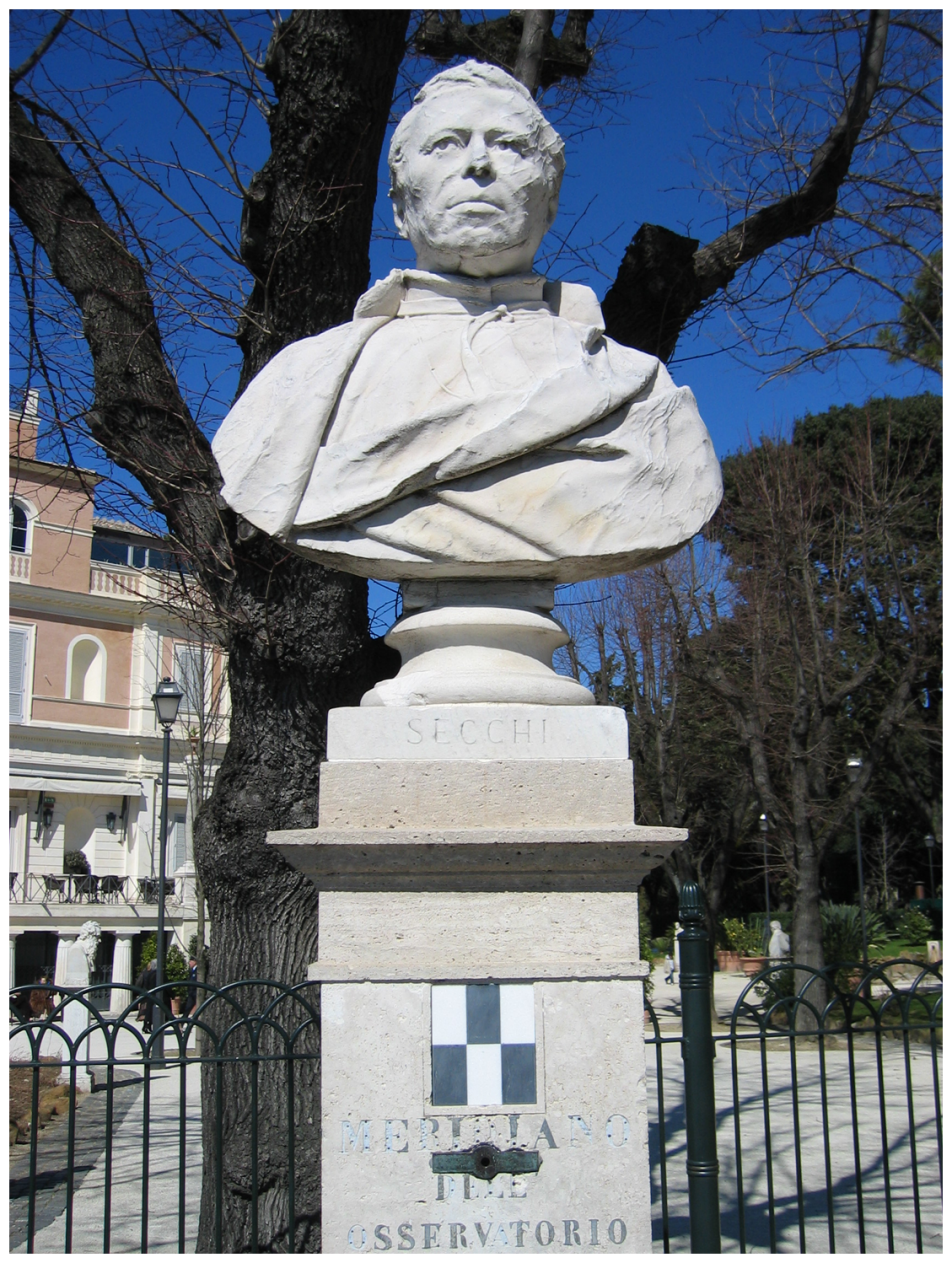
Monument to Pietro Angelo Secchi in the Villa Borghese Park in Rome. Pietro Secchi, who lived in Italy during the 1800s, is recognized as the founder of the method of using a Secchi transparency disk to measure water clarity (Photo by K.E. Webster).

In freshwater ecosystems, the indicator of water quality with the longest history of standardized measurement by scientists and citizens alike is Secchi depth [6]–[8]. The first transparency measurements were made in the ocean during the early 1800s, followed by more careful investigations by Pietro Angelo Secchi in the mid 1800s, which led to his recognition as the method's founder (Fig. 1) [8]. Water clarity is easy and inexpensive to measure using a Secchi disk, and comparisons have shown that citizen-collected Secchi depth readings are nearly identical to those taken by professionals [9], [10]. Consequently, the frequent, citizen-collected Secchi depth readings that are publically available online (e.g., Secchi Dip-In; http://www.secchidipin.org) often span multiple decades for individual lakes and can be used to complement data collected by state and federal agencies.

The U.S. Clean Water Act of 1972 regulates anthropogenic inputs of pollutants to receiving waters [11]. Therefore, Secchi depth records from citizen monitoring programs provide a potentially valuable resource for examining broad-scale responses of lakes to nutrient control efforts instituted in the subsequent 40 years. However, there have been few attempts at compiling and using these data to concurrently analyze spatial and temporal trends at broad scales. Previous studies generally have focused at the scale of a single U.S. state [7], [12], [13].

We compiled citizen-collected, publically available Secchi depth measurements to answer two questions: (1) what are the long-term trends in lake-water clarity across a broad geographic region?; and (2) how do trends differ as a function of spatial location, lake size, and when Secchi depth records were collected? To our knowledge, this is the first study to look at long-term water clarity trends using (1) a broad spatial extent (eight states with a total land area greater than 1.1 million km^2^ in the Upper Midwest region of the United States), (2) a long temporal window (1938–2012), (3) a large number of lakes (>3,000), and (4) publically-available citizen- collected Secchi depth measurements (>140,000). We answer these questions using Bayesian hierarchical modeling, an approach that is useful because it can incorporate disparate data from multiple sources to examine relationships across multiple spatial and temporal extents [14], [15]. Our results can be used as a basis for forming hypotheses to test the mechanisms driving large-scale spatial patterns and temporal trends in water clarity as well as informing local, regional, or federal policies and management actions.

## Methods

### Data Acquisition

**Figure 2:**
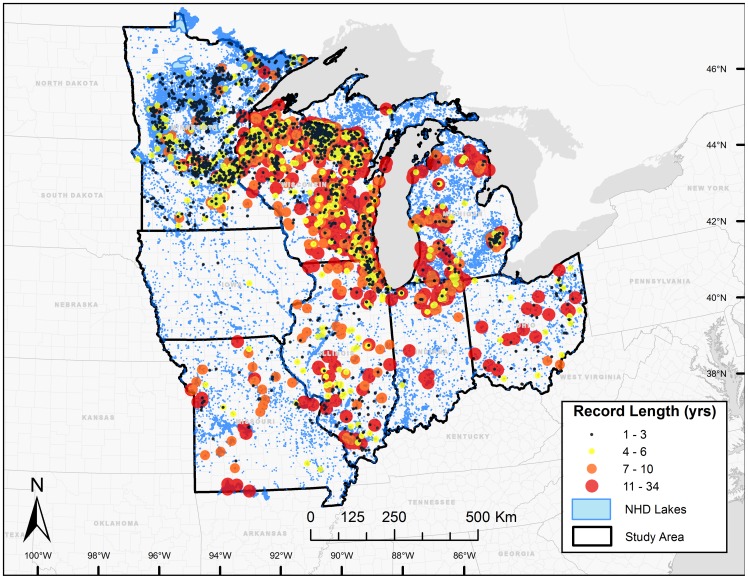
Upper Midwest states in the US and Secchi record lengths (years) for all citizen monitored lakes included in the study. Size and color of symbol designate record lengths. See Table 1 for information on citizen monitoring networks by state.

We obtained 239,741 citizen-collected Secchi depth measurements from monitoring networks in eight states in the Upper Midwest region of the United States (Fig. 2; Table 1) [16]. Observations spanned the time period from 1938–2012 with the oldest records from the state of Minnesota; the other citizen monitoring networks began collecting data during the early 1970s, and the majority (99%) of measurements were taken post-1972. The number of waterbodies monitored varied widely among states, ranging from 5 in Iowa to 1,507 in Wisconsin (Table 1).

**Table 1:** Overview of the data sources and records available from the citizen monitoring networks included in this study.

State	Data Source	# Lakes^1^	# Lakes Monitored	% Lakes Monitored	# Secchi Observations^2^
Illinois	Volunteer Lake Monitoring Program^3^	2,857	303	10.6	40,681
Indiana	Indiana Clean Lakes Program^4^	1,879	95	5.06	8,610
Iowa	Secchi Dip-In Project^5^	1,081	5	0.46	47
Michigan	Michigan Clean Water Corps^6^	6,641	225	3.39	32,222
Missouri	Lakes of Missouri Volunteer Program^7^	1,886	79	4.19	7,581
Minnesota	Citizen Lake Monitoring Program^8^	14,043	961	6.84	13,392
Ohio	Citizen Lake Awareness and Monitoring^9^	1,257	75	5.97	7,819
Wisconsin	Citizen Lake Monitoring Network^10^	5,914	1507	25.5	129,389

Numbers represent totals through 2012.

1 Number of lakes determined from 2007 National Hydrography Dataset (NHD) with surface area equal to or greater than 4 ha.

2 Includes all citizen Secchi observations available from designated monitoring network.

3 http://www.epa.state.il.us/water/vlmp.

4 http://www.indiana.edu/~clp.

5 http://www.Secchidipin.org.

6 http://www.micorps.net/.

7 http://www.lmvp.org.

8 http://www.pca.state.mn.us/index.php/water/water-types-and-programs/surface-water/lakes/citizen-lake-monitoring-program/index.html.

9 http://www.olms.org/citizen-lake-awareness-and-monitoring.

10 http://dnr.wi.gov/lakes/CLMN.

Prior to analysis, Secchi data were put through a series of QA/QC checks, limited to a consistent seasonal window, and summarized as annual averages. First, we converted readings to meters and removed 31 Secchi depth observations that exceeded 30 m. Given the distribution of all Secchi observations (data not shown), these observations appeared to be unrealistic for the region and were likely the result of transcription errors. Second, we limited our analysis to Secchi observations collected during June through August, which removed a source of inter-seasonal variation [17] and generally was the season when most of the monitoring networks collected data (e.g., Secchi Dip-In), providing consistency across sampling networks. Finally, the response variable used for all analyses was average summer Secchi depth calculated for each lake-year, estimated using raw-untransformed Secchi depth values. We also calculated the median sampling date of each lake for use as a predictor variable. For example, for a lake that was continuously monitored by citizens from 1980 through 2000, the median date of the lake record was 1990. Therefore, median sample date represents a surrogate for the time period a given lake was sampled over the entire 1938–2012 span of this study.

Using the latitude, longitude, and unique lake identifiers provided by the individual sampling networks, we merged the study lakes with the high-resolution National Hydrography Dataset (NHD; http://nhd.usgs.gov). We then verified the identity and location (latitude and longitude) of lakes in the dataset and obtained lake surface areas (ha) from the NHD. The final dataset included 148,991 Secchi observations, from which we calculated 21,020 summer lake specific average Secchi depth values for 3,251 lakes that spanned monitoring record lengths from 1 to 34 years (Fig. 2).

### Statistical Analysis

To estimate the overall temporal trend (i.e., trend across all lakes) and assess the variability among lakes in temporal trends, we first fit a hierarchical model with log_e_-transformed Secchi depth as a linear function of year allowing the intercepts, slopes, and model error variances to differ among lakes. The form of the hierarchical model was as follows:
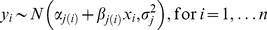


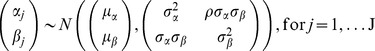


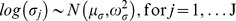
(1)


Where 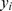 is observation *i* of log_e_-transformed Secchi depth from lake *j*; 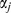 and 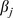 are the intercept and slope (temporal trend) for lake *j*, respectively; *x* is the year covariate (standardized; 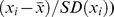 corresponding to the year Secchi observation *i* was measured; 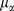 is the population-average intercept and 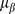 is the population-average slope; 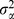 and 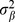 are the variances of the intercepts and slopes, respectively; and 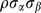 describes the covariance between 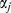 and 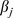, with 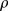 describing the correlation between 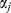 and 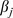. We assumed a normal probability distribution for log_e_-transformed 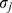 (lake-specific residual variances), with mean 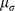 and variance 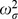. Non-informative normal priors were used for 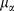, 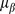, and 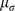 (i.e., 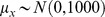). Non-informative uniform priors were used for 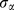, 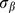, 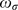 (i.e., uniform distribution on [0,100]), and 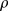 (i.e., uniform distribution on [−1,1]).

Secondly, we fit models that were similar to Eq. 1, but that included predictor variables (i.e., latitude, lake surface area, median sample date) to model variation among lake-specific intercepts, slopes, and residual standard deviations. For example, using latitude (*lat*) as a lake-specific predictor, the model was as follows:
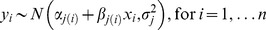


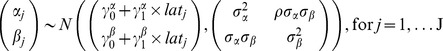


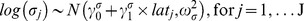
(2)Where 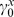 and 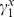 are intercepts and slopes, respectively. Non-informative normal priors were used for 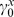 and 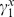 and priors on other parameters were as described above. All covariates were standardized prior to analysis. Using an assessment method similar to classical significance testing, we examined 95% credible intervals (CRIs) for 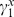 to determine the importance of predictor variables (i.e., examined if 95% CRIs overlapped zero). The models were estimated using Bayesian estimation and the program JAGS was used for all analyses [18]. Three parallel chains were run with different initial values. After discarding the first 10,000 samples, we retained every 3rd sample for a total of 20,000 samples. We examined the scale reduction factor 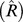, a convergence statistic, for each parameter, trace plots, and plots of posterior distributions to assess convergence.

## Results

**Figure 3:**
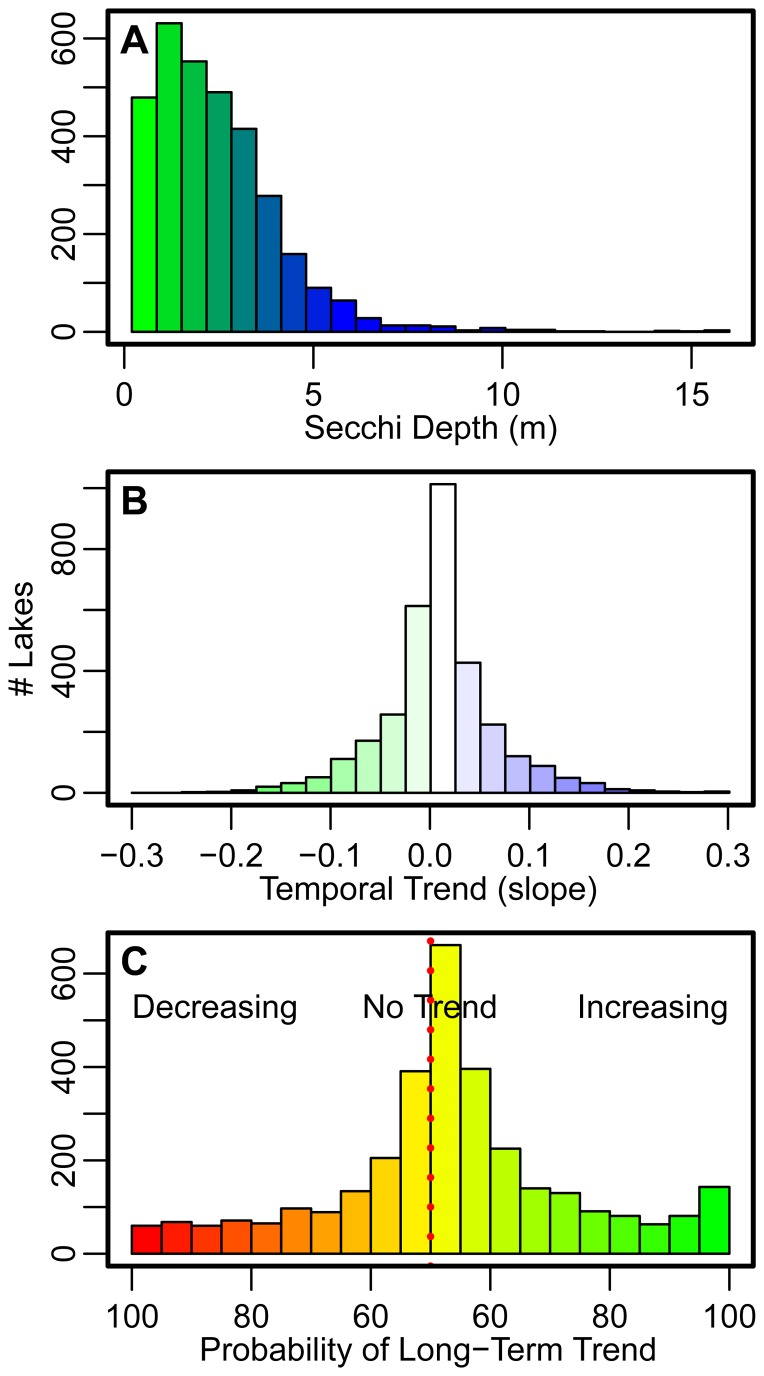
Distribution of lake-specific overall average Secchi depths (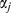 from Eq. 1; A), long-term trends (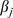 from Eq. 1; B), and probability of lake specific long-term trend (probability of 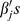 estimated in Eq. 1 of being either <0 or >0; C) based on a hierarchical Bayesian analysis. Long-term trend (B) positive values indicate long-term increases in water clarity (i.e., increased water clarity) and negative values indicate long-term declines in water clarity (i.e., decreased water clarity) for individual lakes. Probability of lake specific long-term trends (C) left of the dotted red line indicate the number of lakes with long-term annual declines in water clarity (Secchi depth becoming shallower) and associated probability of those long-term declines; values to the right indicate the number of lakes with long-term increases in annual water clarity (Secchi depth becoming deeper) and associated probability of those long-term increases.

Average lake specific summer Secchi depth for the 3,251 study lake population covered a wide range from 0.2 to 16 m, with a sample mean of 2.4 m (median 2.1 m; Fig 3A). For a majority of lakes (75%) the year-to-year variability of Secchi depth values within a specific lake was small (coefficient of variation <30%). However, at the population-level, the range was large (coefficient of variation between 6.5 and 184%).

For the **entire lake population**, temporal trends in Secchi depth from 1938 to 2012 were positive (average of all lake specific long-term trends), suggesting an overall increase in water clarity. The posterior estimate for the mean slope, 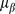, (Eq. 1) was 0.0092, indicating that Secchi depth, on average, has been increasing by 0.92% per year (95% CRI = 0.61–1.79%; Fig. 3B). On an **individual lake** basis, however, there was little evidence for long-term increases or decreases in water clarity for a majority of lakes (Fig. 3C). Of the 3,251 lakes examined, 128 (3.8%) and 224 (6.9%) had a 90% or greater probability of long-term declines or increases in Secchi depth, respectively (i.e., the number of the 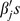 estimated in Eq. 1 had a greater than 90% probability of being either <0 or >0; Fig. 3C).

**Figure 4:**
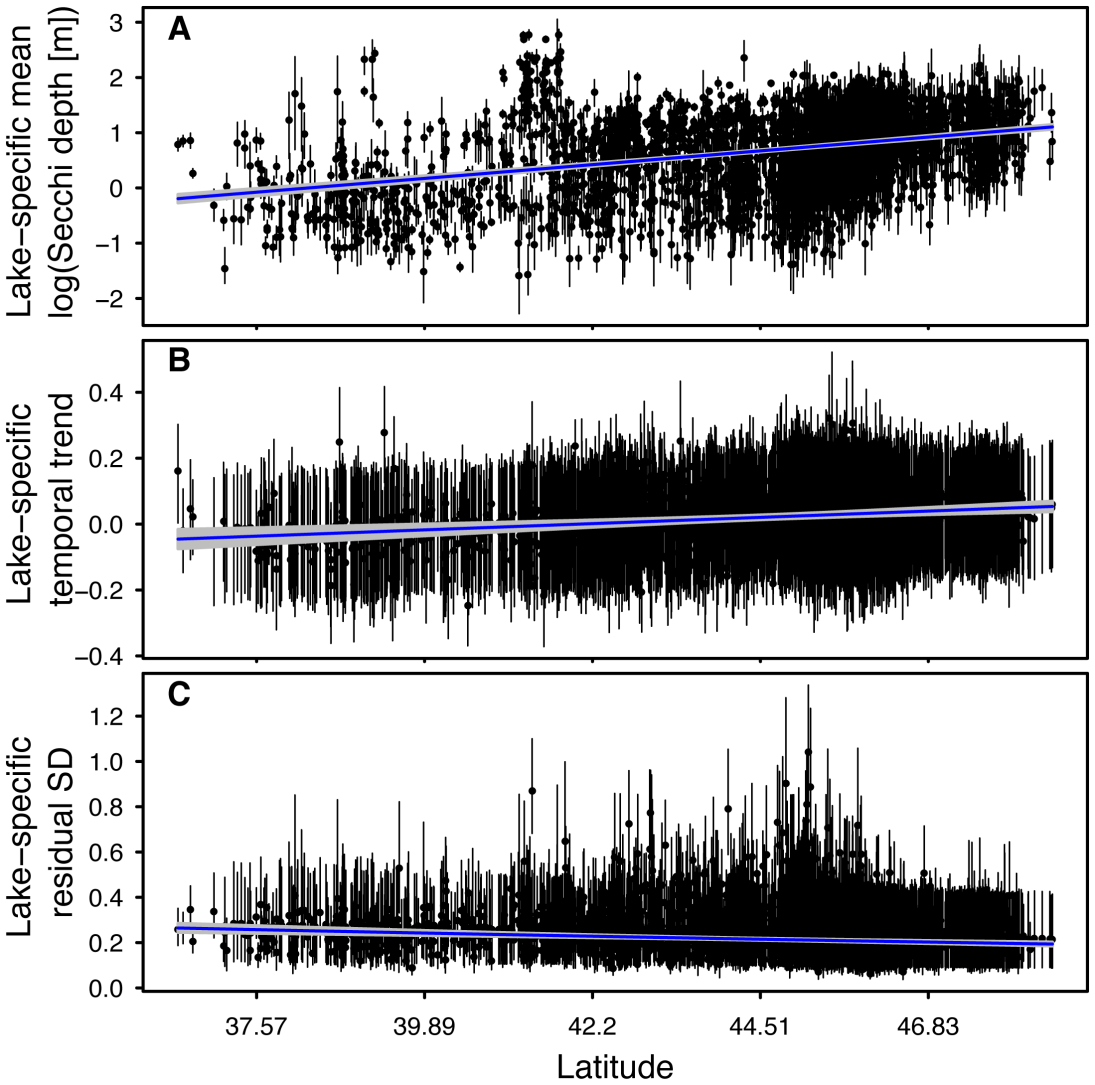
Relationships between average annual summer Secchi depth (A), long-term trend (B), and inter-annual variability (C) and spatial location (latitude) for 3,251 lakes in the Upper Midwest region of the United States. Solid points represent the parameter estimate for each individual lake and vertical lines represent the 95% credible interval of the estimates. Solid blue line represents the trend between each parameter and latitude. Grey shading around the solid blue lines represents the 95% credible interval of the latitudinal trend.

Lake surface area was not related to average annual summer Secchi depth, long-term Secchi depth trend, or inter-annual variability in Secchi depth (posterior means and 95% CRIs for 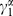 = −0.018 [−0.034, −0.002]; 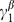 = 0.001 [−0.005, 0.006]; 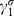 = 0.004 [−0.012, 0.020]). We found relationships between these response variables and the latitude of the monitored lakes (posterior mean and 95% CRI for 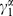 = 0.249 [0.224, 0.274]; 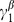 = 0.019 [0.009, 0.029]; 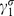 = −0.060 [−0.086, −0.034]). Average annual summer Secchi depth (Fig. 4A) and long-term trend slope (Fig. 4B) increased and inter-annual variability in Secchi decreased (Fig. 4C) across the southern to northern latitudinal gradient (Fig. 2).

**Figure 5:**
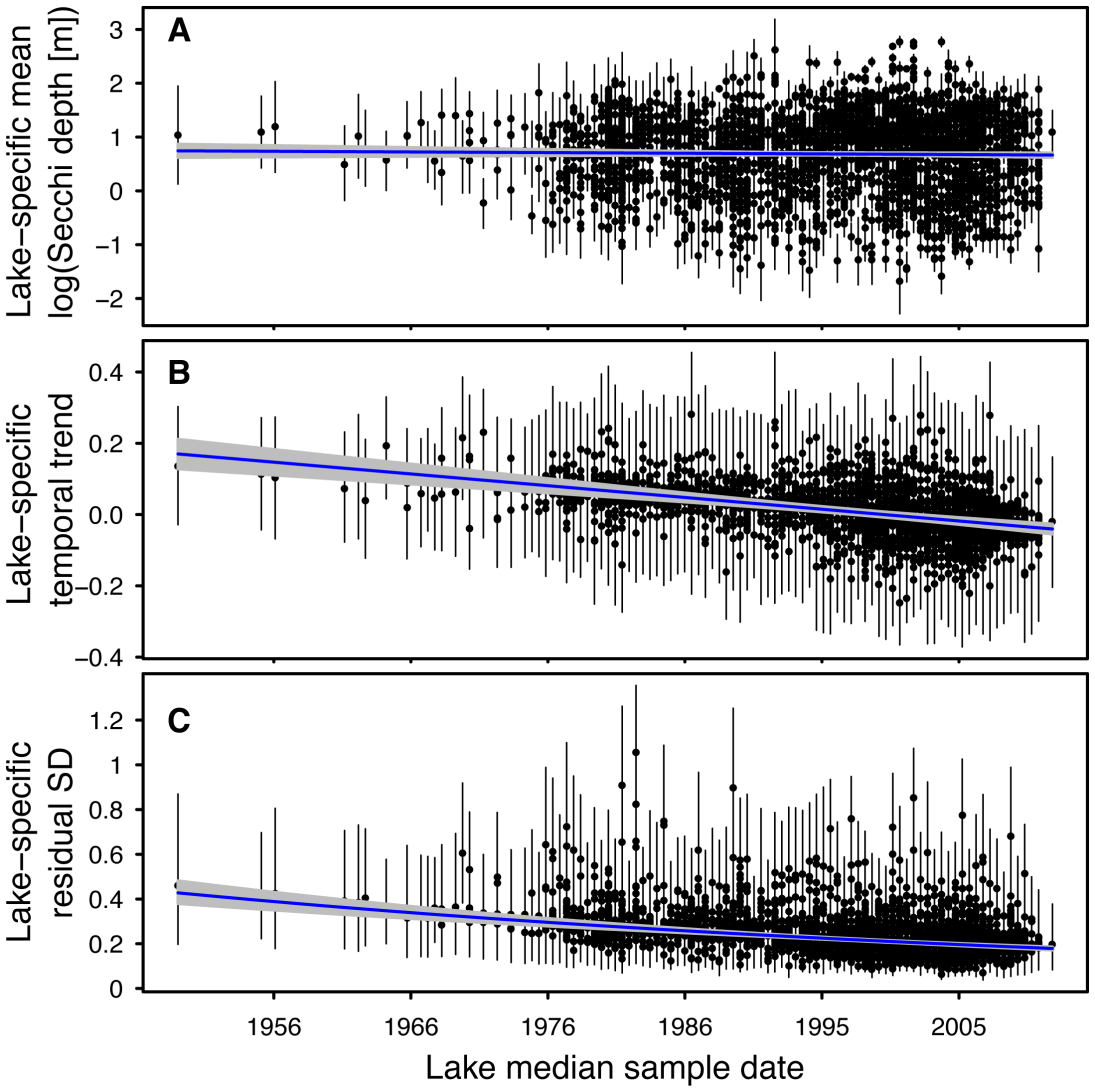
Relationships between average annual summer Secchi depth (A), long-term trend (B), and inter-annual variability (C) and median sample date (see text for details) for 3,251 lakes in the Upper Midwest region of the United States. Solid points represent the parameter estimate for each individual lake and vertical lines represent the 95% credible interval of the estimates. Solid blue line represents the trend between each parameter and latitude. Grey shading is the 95% credible interval of the latitudinal trend.

In addition to detecting spatial patterns in Secchi trends, we found that trends were related to when the data were collected within the multi-decadal time interval of 1938–2012 (i.e., the median year of sample collection). Median sample date was negatively related to both long-term Secchi depth trend and inter-annual variability (posterior mean and 95% CRI for 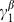 = −0.033 [−0.043, −0.023]; 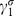 = −0.137 [−0.164, −0.108]; Fig. 5B, C). These results suggest that historical records were characterized by more positive annual Secchi depth trends relative to records collected more recently (Fig. 5B) and that inter-annual variability of Secchi depths was greater historically than what is observed from more current records (Fig. 5C). There was no evidence that relationships were due to differences in the water clarity of lakes being monitored by citizen volunteers over time, which has not changed (posterior mean and 95% CRI for 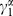 = −0.012 [−0.043, 0.017]; Fig. 5A).

## Discussion

This study relied solely on citizen monitoring data to assess long-term water clarity (quantified as Secchi depth) trends and their relationships with spatial location, size of lake monitored, and time period monitoring took place. Overall, water clarity has been increasing slightly for the **entire population** of monitored lakes (average trend slope [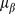] > zero). Eleven percent of lakes have strong trends in water clarity (90% probability slope is different than zero), with two-thirds corresponding to increased water clarity. We observed compelling spatial and temporal patterns with respect to the water clarity, long-term trends, and inter-annual variability of water clarity. Other studies have observed similar patterns in citizen monitored and remotely sensed Secchi depth [12], [13] but over no more than two decades. We are unaware of any studies conducted with citizen-collected Secchi data at the spatial and temporal scales considered in this study.

Previous analyses of satellite-derived long-term Secchi depth patterns in the study region found that on average water clarity trends have been relatively stable (Minnesota) [13], [19] or increasing (Wisconsin) [12]. We observed that clarity is increasing on average by about 1% per year for the **entire population of lakes** included in this study. In large part, the long-term average increases in water clarity reported here were driven by data from lakes in Minnesota and Wisconsin, which comprise 76% of total number of lakes in the dataset (30% and 46% respectively). Excluding these two states, the average annual increase in clarity was negligible (0.17%). Interestingly, we found larger annual increases in Minnesota (1.59%) relative to Wisconsin (0.88%), which is contrary to the satellite-derived results that suggested relatively stable long-term patterns in Minnesota and more substantial increases in Wisconsin [12], [13]. It is important to note, however, that the different approaches taken for these studies (e.g., citizen-collected Secchi data or satellite derived data, frequentist or Bayesian statistical approaches) to identify long-term trends are not suggesting drastically different patterns at regional scales (e.g., increasing versus decreasing), but rather subtle differences in the magnitudes of overall changes.

On an **individual lake** basis, results from our large multi-state study (e.g., Fig. 3) are generally consistent with small-scale lake-specific studies [7], [20], [21]. The analytical approaches employed in our study, however, provided an opportunity to combine space and time to analyze temporal trend patterns across a broad spatial extent. A majority of our lakes exhibited relatively stable (i.e., no significant long-term directional trend) water clarity with minimal inter-annual variability, whereas a small percentage of lakes (10.7%) exhibited strong evidence of long-term directional trends. These results are similar in magnitude to a recent analysis of satellite derived Secchi depths for >10,000 lakes, which found significant directional trends over 20 years in 10.8% of all Minnesota lakes [19]. Prior state-wide analyses of citizen volunteer data have also found similar patterns in water clarity over time. For example, over 50, 80, and 60% of the 50–122 lakes monitored by citizens in Minnesota, Wisconsin, and Michigan, respectively, showed no trend in water clarity over time [7], [20], [21]. Increasing trends in water clarity were found for 36, 31, and 10% of lakes in these three states, respectively, with a much smaller percentage of lakes in these studies showing decreasing water clarity over time [7], [20], [21]. Our study of a much larger number of lakes located across a very large spatial extent corroborates these previous studies and demonstrates that (1) most citizen monitored lakes in the Upper Midwest of the United States have had a relatively stable long-term water clarity record and (2) long-term increases in water clarity for individual lakes occur more frequently than do declines.

Some of the most compelling results emerging from our analyses are the relationships between long-term water clarity trends and the spatial location of lakes. Our results suggest that lakes situated at more southern latitudes tended to have trends of long-term decline in water clarity, whereas lakes situated at more northern latitudes had more of an overall shift towards long-term increases in water clarity (Fig. 5B). Although the water clarity patterns presented here were correlated with latitude, we suspect that these patterns were likely more directly influenced by a secondary, confounded, variable such as land use/cover [7], [22] or climate [23]. For example, an analysis of satellite water clarity data from Minnesota over the last 20 years found similar patterns emerged at the state-level that were correlated with urban/agriculture (decreasing water clarity) and forested (increasing water clarity) land use types [19]. There is substantial spatial heterogeneity across our study extent, with extremes represented by large differences between the southern-most and northern-most regions in local and regional land use/cover patterns, as well as the underlying geology and soil patterns that are confounded with those human land uses. Consequently, understanding these regional water clarity patterns requires information about physical, chemical, and biological lake properties as well as data on watershed and regional landscape features at broad spatial and temporal scales [24]. However, a broad-scale, multi-thematic database containing such variables does not exist.

**Figure 6:**
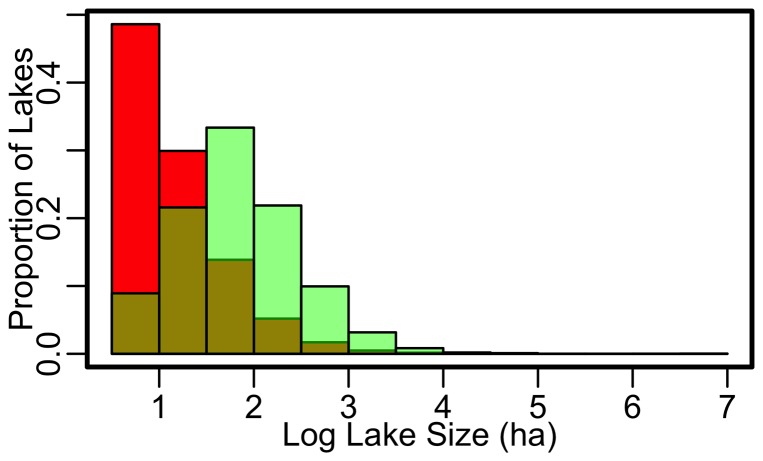
Distribution of lake sizes (surface areas) >4 hectare in the study region based on 2007 National Hydrography Dataset (red) relative to the distribution of lake sizes included in citizen science monitoring networks (green). Data are Log_10_ transformed.

We also examined water clarity trends across the time period that encompassed passage of the U.S. Clean Water Act, leading to the expectation that an increase in water clarity would have occurred during the past 30–40 years. Our study did not find this pattern, at least not at the aggregate scale and for this population of lakes; in fact, the analyses provided evidence of stronger increases in the early part of the record. There are three possible reasons for this result. First, the data in our study are not continuous for all lakes pre- and post-Clean Water Act. Second, it is critical to acknowledge that lakes monitored by citizens are likely not representative of all lakes within the study extent. Although a substantial number of water clarity measurements have been made, only a fraction of all lakes in each state had water clarity actively monitored by citizen scientists (Table 1). Additionally, the distribution of surface areas of citizen-monitored lakes diverged from that of the entire distribution of lakes in our study extent (Fig. 6). The lakes that citizens monitor are disproportionately larger than would be expected based on the size distribution of lakes in general [25]. Similar biases towards overrepresentation of large lakes have been observed when examining the types of lakes monitored by state agencies [26]. We believe that this is the largest dataset of citizen-monitored Secchi data to be analyzed to date and suspect this bias may also exist for datasets from beyond our study region. The patterns presented here are characteristic of the suite of lakes included in our dataset and, thus, they may not be representative of all lakes in our study region or those beyond it. Consequently, careful consideration needs to be made should these results be extrapolated to lake populations other than the those considered in this study [26]. Third, water clarity is affected by a number of natural features that alter light attenuation including dissolved organic carbon, chlorophyll, and suspended sediments [17] and is altered by human-induced nutrient inputs [27], invasion by non-native species (e.g., zebra mussels) [28], acidification [29] and climate change (browning of waters) [23]. The complex set of factors affecting lake water clarity, many of which are correlated, makes it difficult to interpret the causes of trends in water clarity and has, perhaps, led to Secchi depth being considered a relatively coarse indicator of overall water quality. However, its use as an approximation of water quality status [30] and as a measure of the reliability of satellite-derived water clarity data [13], combined with ease of collection by citizen scientists, suggests this 150-year measure of water clarity will continue to provide valuable insights into the condition of lake ecosystems.

## Conclusions

Although there has been a long history of examining water clarity trends and patterns augmented with citizen collected data, the results presented here incorporate over 140,000 citizen monitoring water clarity values, extending back to 1938, collected at greater spatial and temporal scales than any previous study we are aware of. It is impossible for aquatic management organizations to monitor all lakes all the time; citizen water clarity data have been critical in augmenting traditional agency monitoring efforts and provide vital data for identifying and understanding water quality patterns at broad spatial and temporal scales (e.g., calibration of satellite-derived water clarity estimates). Here, we demonstrate the effectiveness of citizen data for quantifying long-term water clarity trends in individual lakes and identifying compelling population-level patterns in lake water clarity at the macro-scale. In an era of limited resources and during a push toward applying data-intensive science to environmental management and policy, the importance of citizen data such as these is likely to increase. Therefore, there is a clear need to continue to learn how to effectively leverage these types of data in the future.

These data needs coincide with the increasing recognition that stressors occurring at local, regional, and global scales interact to influence ecological patterns and processes from local to macro-scales [24]. At the scale of an **individual lake**, our data indicate that a majority of lakes in the Upper Midwest region have had relatively stable long-term water clarity patterns. However at the **regional scale**, we observe emerging macro-scale patterns in long-term water clarity trends and inter-annual variability. Understanding the linkages between the multi-scale water clarity patterns presented here and the factors like climate, land use/cover, and biotic interactions that have been shown to influence water clarity [7], [23], [31] have been elusive. Although citizen science affords a unique opportunity to obtain data such as water clarity at unprecedented spatial and temporal scales, we generally lack the large, multi-thematic data necessary to explain these patterns and fully apply a macrosystems ecology approach. Consequently, there is a need to compile multi-thematic data across multiple spatial and temporal scales in order to link patterns observed at local and regional scales to processes occurring across the landscape. We believe that collaborations among citizens, research scientists, and local, state and national agencies are important for developing the data sources and analytical tools necessary to move beyond identifying patterns toward an understanding of the critical factors influencing macro-scale patterns such as those shown here for lake water clarity.
